# Pembrolizumab-Induced Choriocapillaritis and Orbital Inflammation: A Case Report

**DOI:** 10.7759/cureus.98725

**Published:** 2025-12-08

**Authors:** Oumaima El Korno, Zineb Hilali, Taha Boutaj, Hassna El Ghazi, Saad Benchekroun, Samira Tachfouti, Abdellah Amazouzi, Lalla Ouafa Cherkaoui

**Affiliations:** 1 Ophthalmology, Centre Hospitalo-Universitaire Ibn Sina, Hôpital des Specialités, Rabat, MAR; 2 Ophthalmology, Mohammed V University, Hôpital des Specialités, Rabat, MAR

**Keywords:** autoimmune choriocapillaritis, immune checkpoint inhibitors, immune-mediated ocular inflammation, medical oncology, melanoma, ocular immune-related adverse events, orbital inflammatory disease, pembrolizumab

## Abstract

Immune checkpoint inhibitors (ICIs) such as pembrolizumab have significantly extended patient survival in metastatic melanoma; however, they may rarely induce heterogeneous and potentially vision-threatening ocular immune-related adverse events (irAEs). We report a case of a 52-year-old man with metastatic superficial spreading melanoma who, after two years of pembrolizumab therapy, presented with bilateral ocular redness and photopsias. Visual acuity was preserved in both eyes. Fundus examination showed multifocal pigmentary changes. Fundus autofluorescence (FAF) revealed corresponding hypoautofluorescent spots without macular involvement, and indocyanine green angiography (ICGA) demonstrated multifocal hypercyanescent lesions consistent with choriocapillaritis. Orbital MRI demonstrated extraocular muscle enlargement and intraconal fat inflammation suggestive of posterior orbitopathy, without exophthalmos or optic nerve compression. Given the excellent systemic response and preserved vision, a multidisciplinary team elected to continue pembrolizumab, initiate topical corticosteroids, and ensure close ophthalmologic follow-up. Systemic corticosteroids were reserved for potential progression. This rare association of multifocal choriocapillaritis and posterior orbitopathy under pembrolizumab underscores the expanding spectrum of ocular irAEs. Importantly, when findings are mild and vision is preserved, pembrolizumab may be continued with local treatment. Early recognition and close collaboration between ophthalmology and oncology are essential to prevent irreversible visual morbidity while maintaining optimal oncologic outcomes.

## Introduction

Immune checkpoint inhibitors (ICIs) such as anti-programmed cell death 1 (anti-PD-1; pembrolizumab, nivolumab), anti-programmed cell death-ligand 1 (anti-PD-L1), and anti-cytotoxic T-lymphocyte-associated protein-4 (anti-CTLA-4) agents have reshaped the management of advanced melanoma by restoring antitumor immune activity. Since their first approval in 2011, their indications have progressively expanded to multiple malignancies, including non-small cell lung cancer, urothelial carcinoma, cutaneous malignancies, and lymphomas [1,2].

By enhancing T-cell activation, ICIs can induce immune-related adverse events (irAEs) affecting multiple organs. Ocular irAEs are uncommon but may be vision-threatening, with reported manifestations ranging from ocular surface disease and uveitis to orbital inflammation and posterior segment involvement such as serous retinal detachment, birdshot-like chorioretinopathy, and optic neuritis [3-5]. Because most published data consist of isolated reports, each additional case contributes to improved recognition and management of these reactions.

We present a case of pembrolizumab-associated multifocal choriocapillaritis with posterior inflammatory orbitopathy in a patient with advanced cutaneous melanoma. This uncommon association not only broadens the spectrum of ICI-related ocular immune-related adverse events but also highlights the therapeutic dilemma of whether to continue or interrupt immunotherapy, a decision that must be individualized according to the severity of ocular involvement and the overall oncologic benefit.

## Case presentation

A 52-year-old man with a history of superficial spreading melanoma was referred to our department for bilateral ocular redness and photopsia. His medical history was significant for prior surgery for an intramucosal adenocarcinoma of the colon, followed by the diagnosis of cutaneous melanoma that subsequently progressed to bone and pulmonary metastases. Given the extent of metastatic disease, he was started on immunotherapy with pembrolizumab (Keytruda®), which resulted in an excellent systemic response on follow-up imaging, with significant regression of metastatic lesions on PET-CT scans. Pembrolizumab remained the only effective systemic therapy available for his disease.

**Figure 1:**
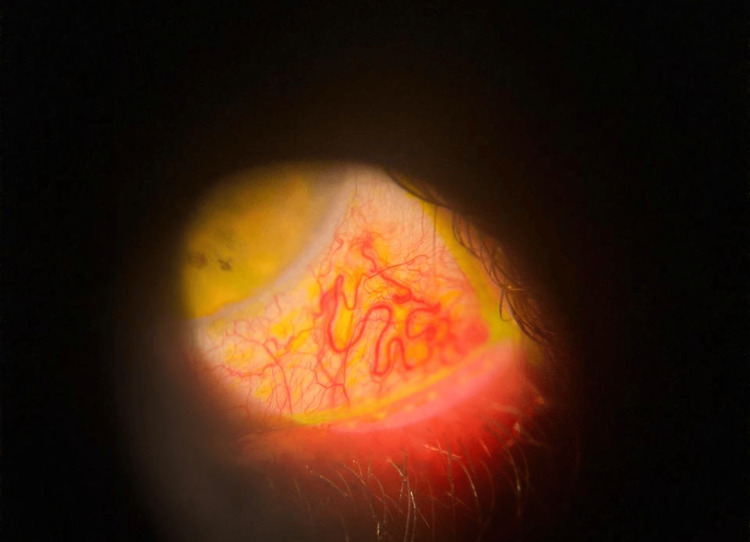
Slit-lamp photograph showing sectoral episcleritis with dilated and tortuous episcleral vessels.

After approximately two years of continuous pembrolizumab therapy, he developed new-onset bilateral ocular symptoms. Best-corrected visual acuity was 6/10 in the right eye, reduced due to astigmatism induced by a nasal pterygium, and 10/10 in the left eye. Pupils were equal and reactive in both eyes, with no relative afferent pupillary defect. Ocular motility was full in all directions of gaze, without diplopia or pain on eye movements. Anterior segment examination revealed dilated and tortuous episcleral vessels, consistent with episcleritis, without corneal involvement or anterior chamber reaction (Figure 1).

**Figure 2:**
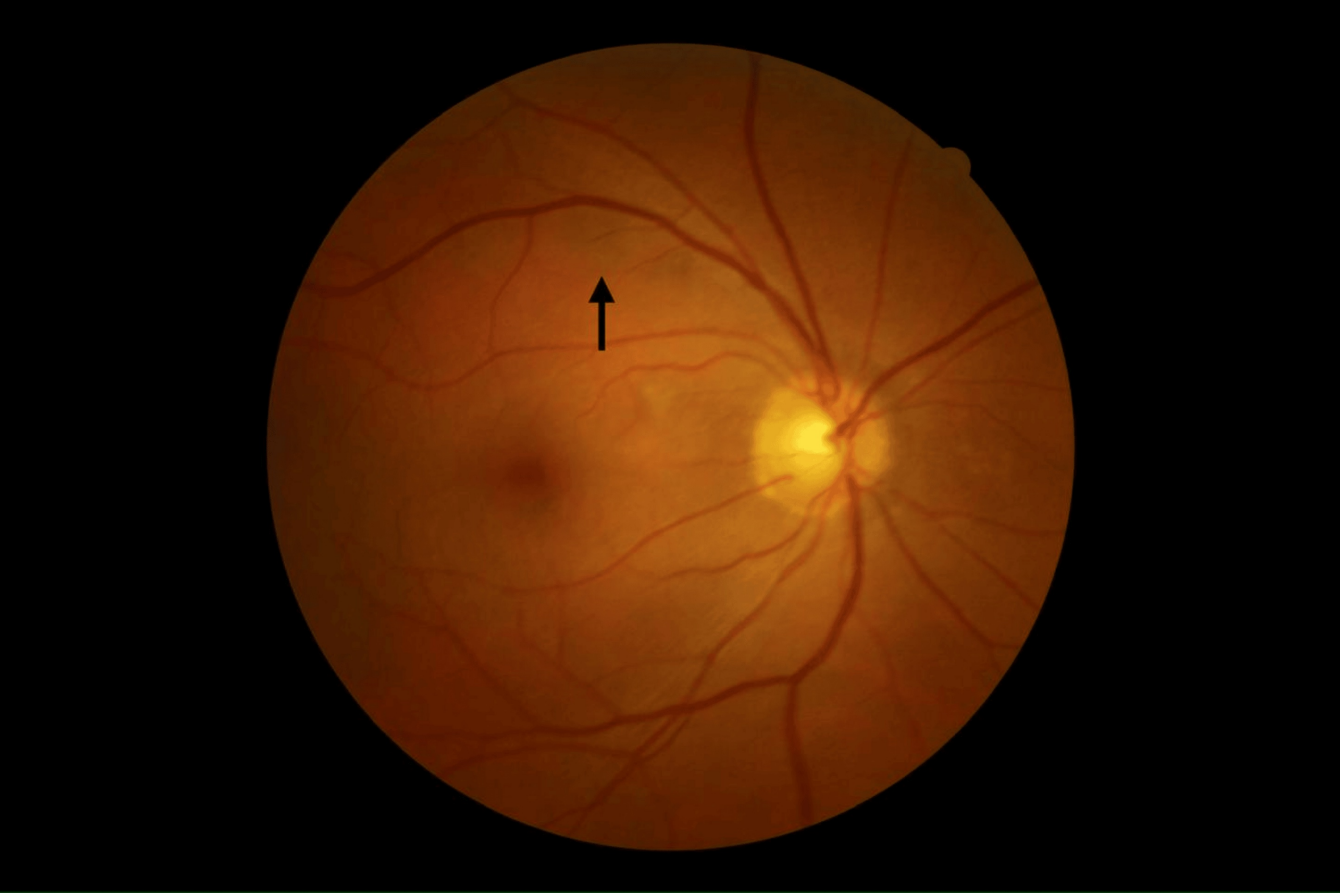
Fundus photography of right eye showing chorioretinal inflammatory lesions (arrow) located at the posterior pole.

**Figure 3:**
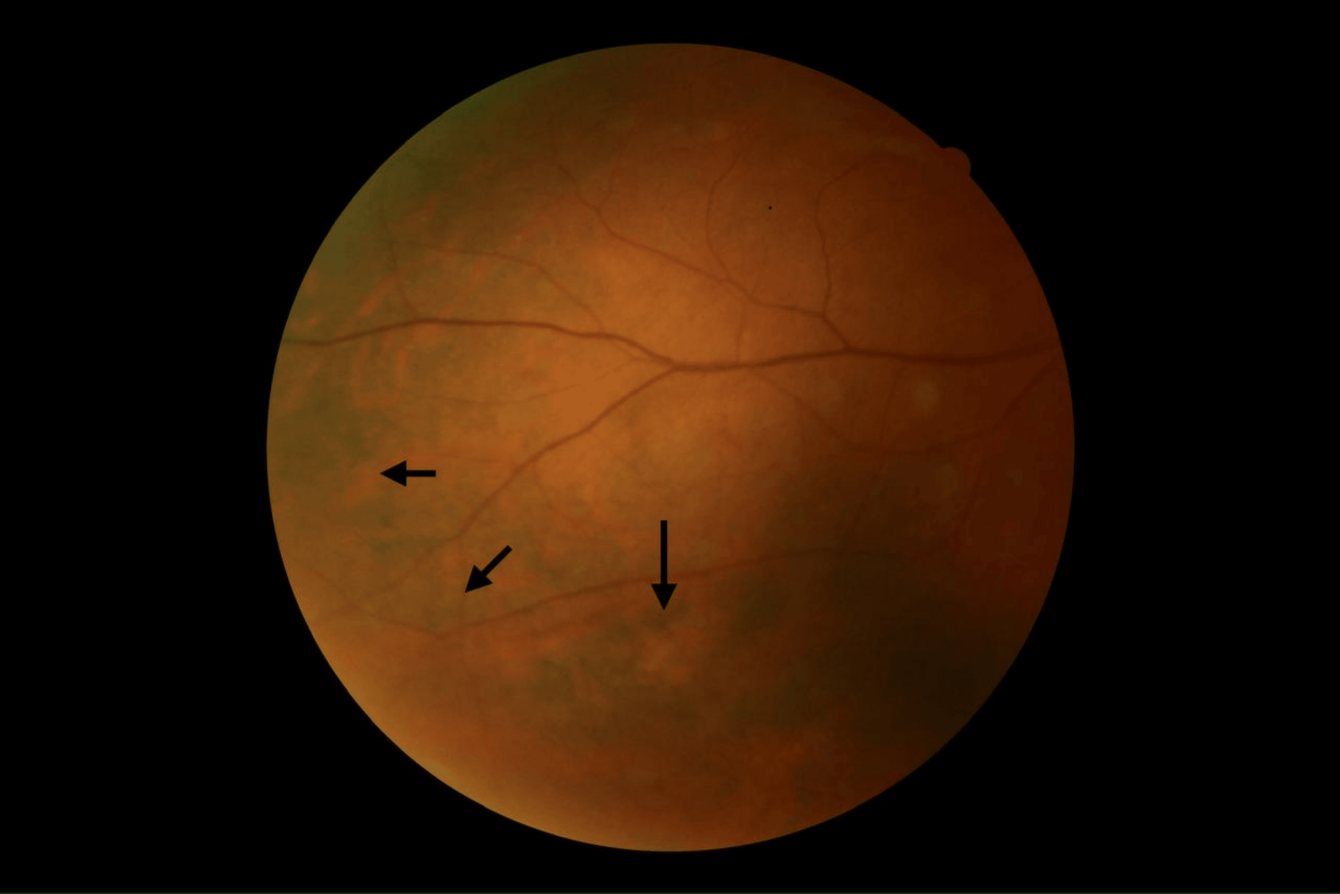
Fundus photography showing chorioretinal inflammatory lesions (arrows) located at the periphery of the retina.

Color fundus photography revealed posterior chorioretinal pigmentary changes in both eyes, scattered at the posterior pole (Figure 2) and the periphery (Figure 3), with a normal macula and optic disc.

**Figure 4:**
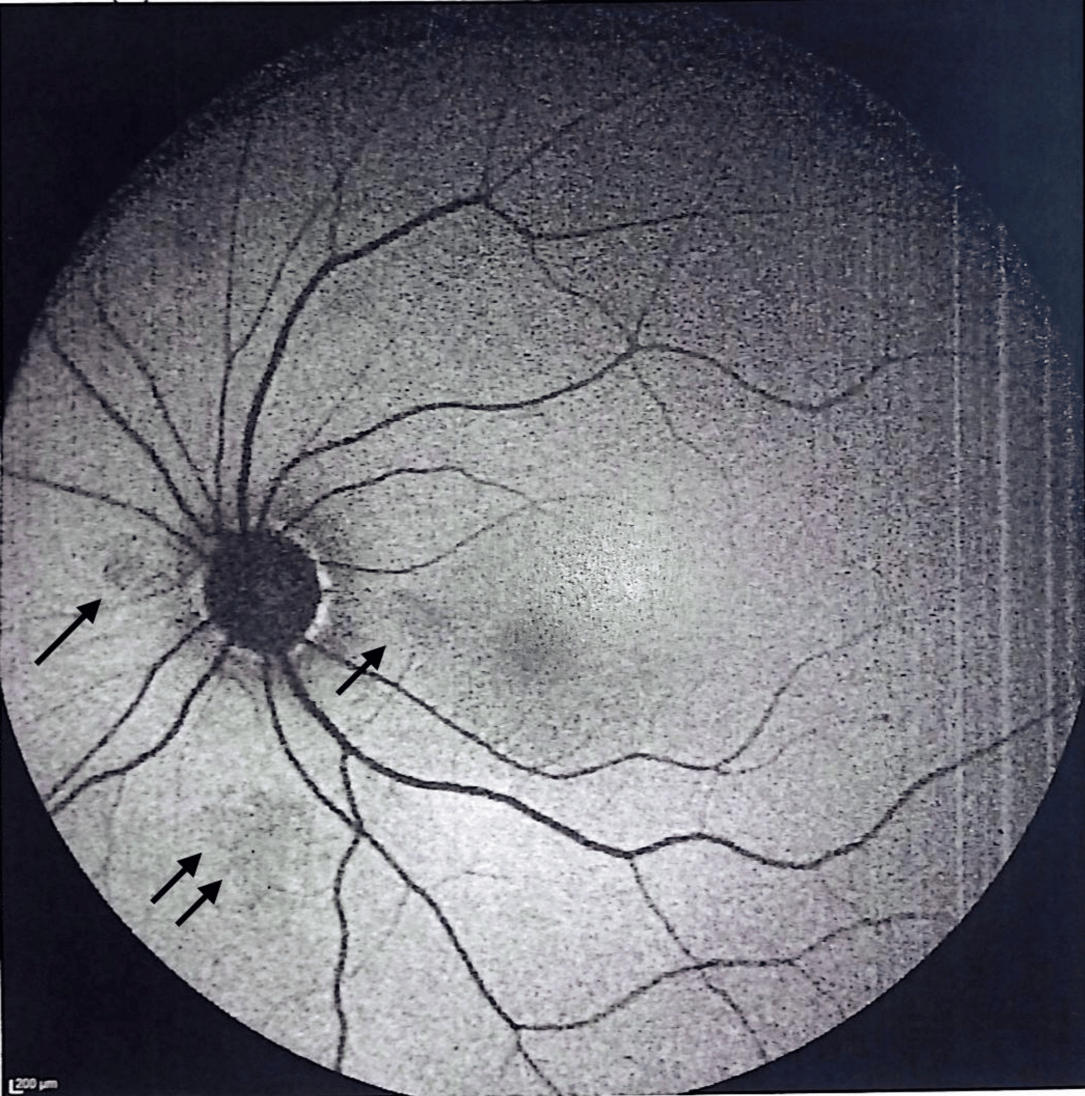
FAF imaging revealing patchy hypoautofluorescent areas (arrows) in the posterior pole, reflecting focal RPE dysfunction. FAF: fundus autofluorescence, RPE: retinal pigment epithelium.

Fundus autofluorescence (FAF) revealed numerous discrete hypo-autofluorescent spots, indicating areas of retinal pigment epithelium (RPE)/choriocapillaris dysfunction with preservation of the fovea (Figure 4).

**Figure 5:**
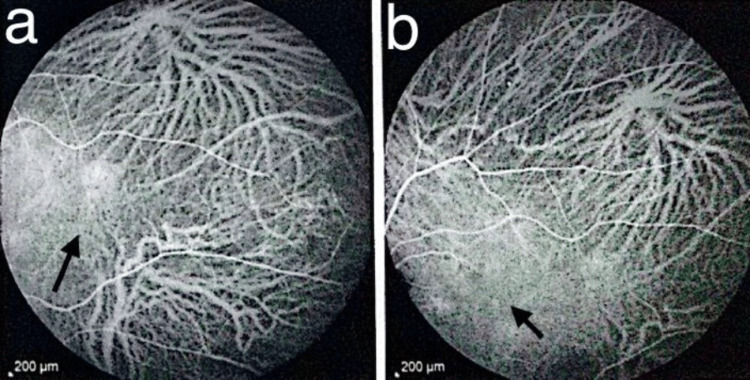
Indocyanine green angiography findings. (a) Early-phase ICGA showing a focal hypercyanescent inflammatory lesion at the posterior pole (arrow). (b) Late-phase ICGA demonstrating persistence of the same hypercyanescent lesion (arrow), marking the area of interest. ICGA: Indocyanine green angiography.

**Figure 6:**
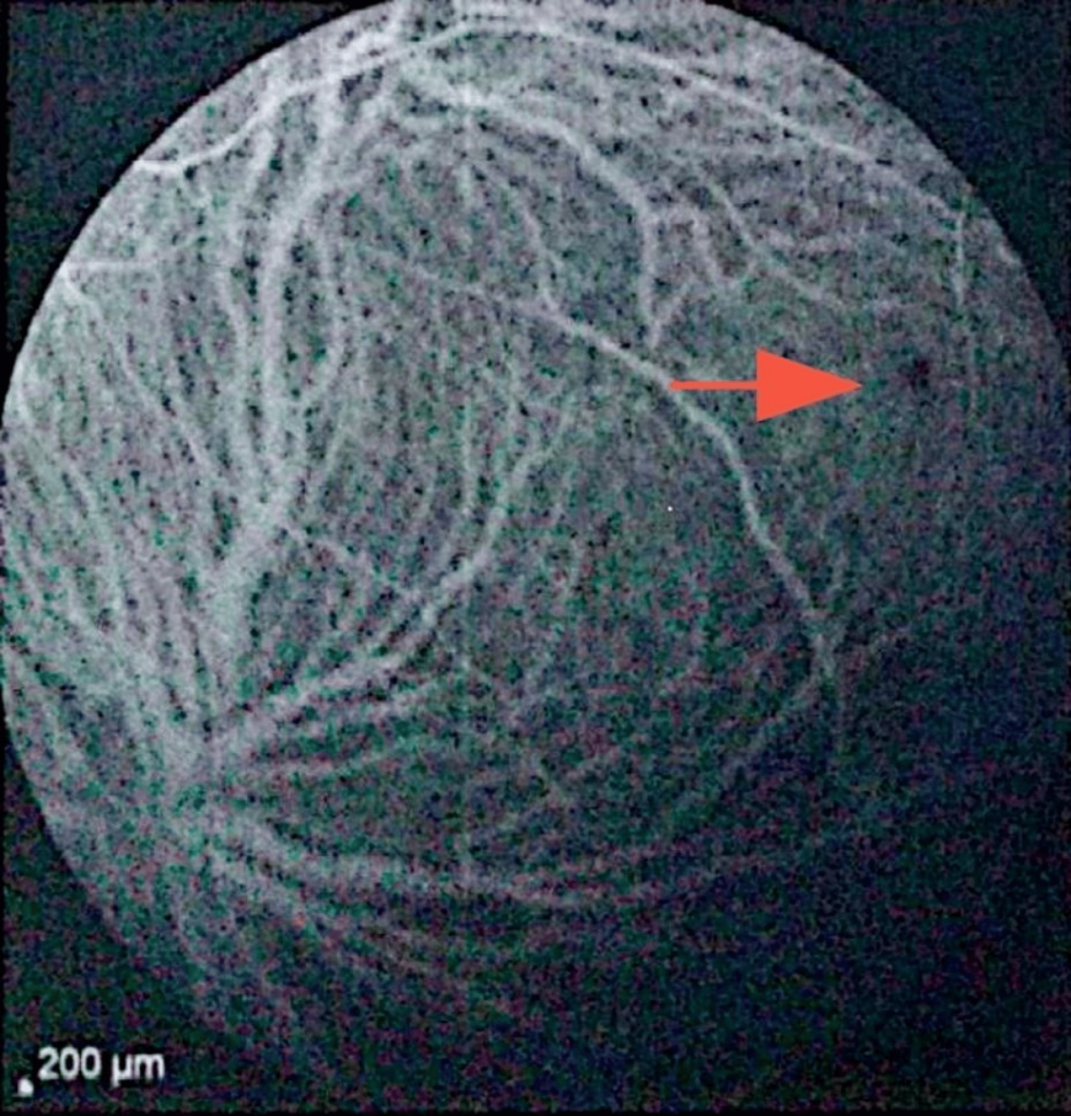
ICGA demonstrated a small punctate hypocyanescent foci (red arrow). This is suggestive of focal choriocapillaris hypoperfusion within the inflammatory process. ICGA: indocyanine green angiography.

Indocyanine green angiography (ICGA) demonstrated well-defined hypercyanescent lesions visible from early phases and persisting in late phases, together with punctate hypocyanescent foci, the latter confirming choriocapillaris non-perfusion consistent with an immune-mediated choriocapillaritis (Figures 5, 6).

**Figure 7:**
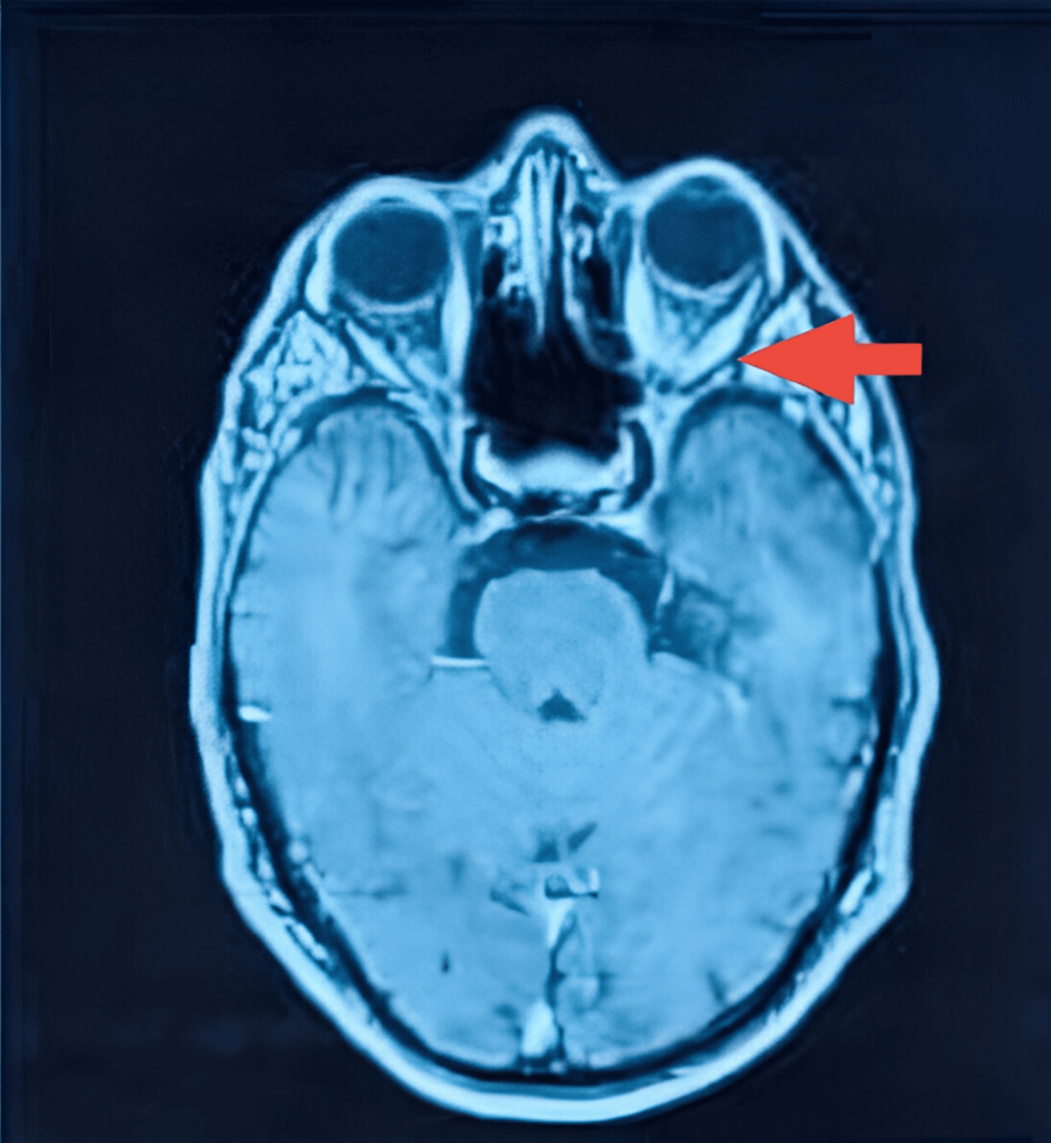
Axial T1-weighted MRI after gadolinium injection. The image shows bilateral enlargement and contrast enhancement of the extraocular muscles(red arrow), with associated enhancement of the intraconal fat. The tendon insertions are spared.

**Figure 8:**
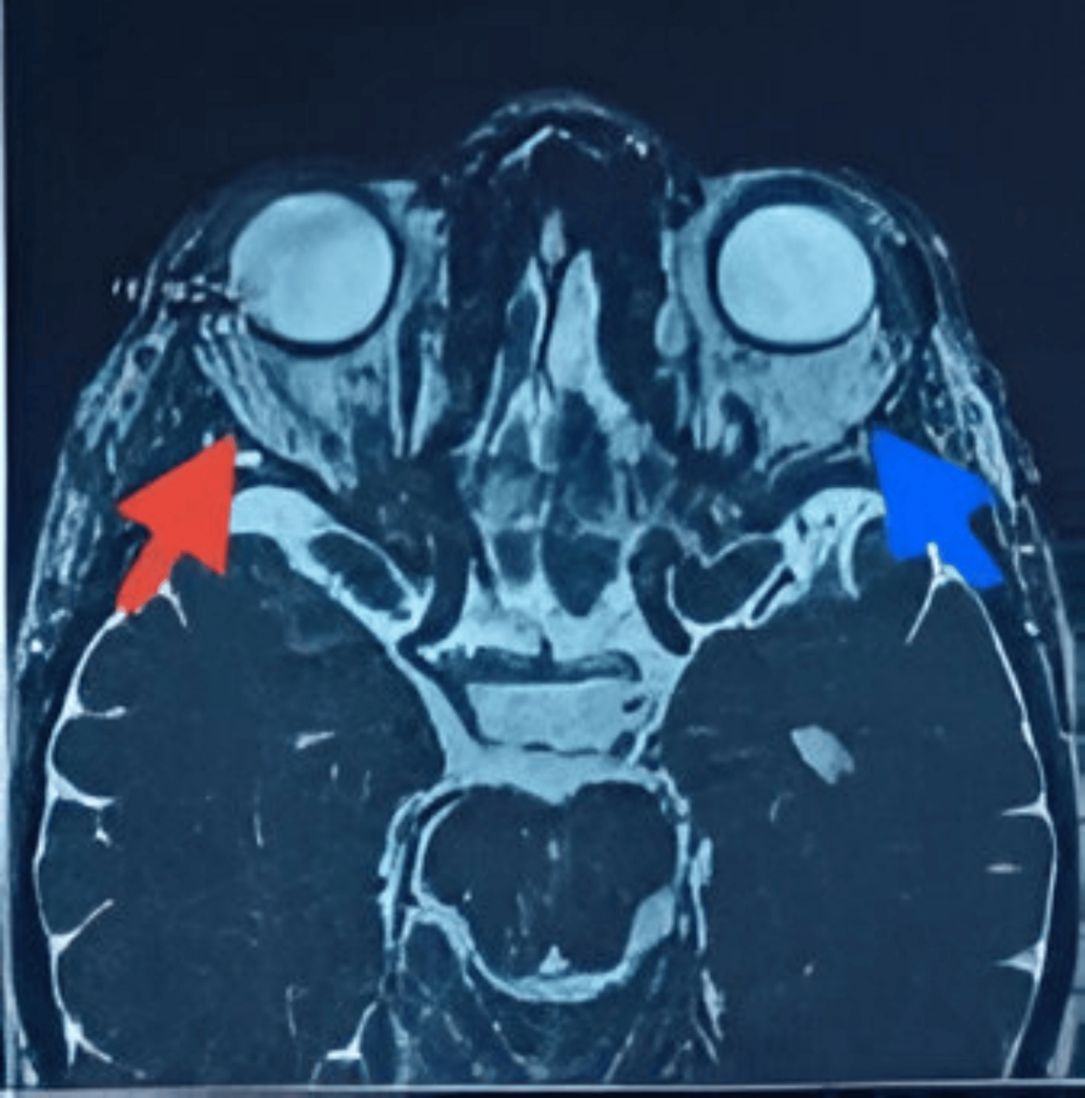
Axial T2-weighted fat-suppressed image. Image is Demonstrating fusiform enlargement of the extraocular muscles with marked T2 hyperintensity (red arrow), more pronounced on the left. The intraconal fat shows diffuse inflammatory hyperintensity (blue arrow), consistent with active orbital inflammatory involvement.

Spectral-domain optical coherence tomography (OCT) of the macula was normal in both eyes, with preservation of the foveal contour and no intraretinal or subretinal fluid. Orbital MRI showed bilateral enlargement of the extraocular muscles with contrast enhancement after gadolinium injection, together with inflammatory intraconal fat infiltration appearing hyperintense on T2-weighted images, compatible with posterior inflammatory orbitopathy, with no signs of exophthalmos or optic nerve compression (Figures 7, 8).

Infectious, metastatic, and alternative inflammatory causes were ruled out based on clinical evaluation, multimodal ocular imaging, and systemic oncologic assessment. A joint ophthalmology-oncology decision was taken. The patient had undergone extensive metastatic staging prior to initiating pembrolizumab, and his recent PET-CT scans demonstrated an excellent therapeutic response with no evidence of active systemic disease. Pembrolizumab remained the only effective oncologic option for his melanoma, and interruption of treatment was considered to carry a significant risk of metastatic recurrence or progression.

Given the preserved visual acuity, the absence of macular involvement, and the mild severity of the ocular findings, the multidisciplinary team elected not to discontinue pembrolizumab. Although orbital imaging showed bilateral extraocular muscle involvement, there were no clinical signs of compressive optic neuropathy, diplopia, or significant motility impairment. In this context, the orbital inflammation was considered mild and non-vision-threatening. 

The patient was started on topical corticosteroid therapy for the episcleritis, and a strategy of close ophthalmologic follow-up was chosen, with the possibility of initiating systemic corticosteroids or additional immunomodulatory treatment should inflammation worsen. This approach aimed to maintain oncologic control while ensuring visual preservation through tight monitoring.

## Discussion

Immune checkpoints are physiological regulators of the immune system that restrain T-cell activation to preserve immune balance and prevent collateral tissue injury. PD-1 (also called PDCD1 or CD279) is expressed on activated T cells, while its major ligand, PD-L1, is expressed on various cells, including tumour cells. Engagement of PD-L1 with PD-1 delivers an inhibitory off signal that suppresses T-cell activity and enables tumour cells to escape immune destruction. Immune checkpoint inhibitors (ICIs), such as anti-PD-1 (pembrolizumab, nivolumab), anti-PD-L1, and anti-CTLA-4 (ipilimumab) antibodies, block these pathways and restore antitumor cytotoxic activity [1,2].

While ICIs have revolutionized melanoma therapy, they may also induce ocular irAEs. Although uncommon, these events are increasingly recognized and can affect both anterior and posterior segments [3,4]. In the eye, disruption of immune homeostasis may allow autoreactive T cells to target the choroid, retina, or extraocular muscles, with the choroid particularly vulnerable due to its dense vascular and immune network [5].

Review data show that while anterior segment disorders (dry eye, blepharitis, conjunctivitis) are most frequently reported, posterior segment involvement (retina, choroid) and orbital disorders, though less common, are increasingly documented [3-5]. In a systematic review including 179 studies and 290 cases of ocular adverse events associated with ICIs, uveitis was the most common adverse event (n=134; 46.2%), followed by neuro-ophthalmic disorders (n=71; 24.5%). Adverse events affecting the orbit and cornea were reported in 33 (11.4%) and 30 (10.3%) cases, respectively, and retinal involvement in 26 cases [4].

Another comprehensive overview of ocular immune-related adverse events (OirAEs) confirmed that the most frequently reported adverse events associated with the anterior segment include dry eye disease and uveitis [5]. A case series of 28 patients (36 ocular irAEs) found that anti-PD-1 agents were responsible for 57% of events, with anterior uveitis being the most common (44%), followed by panuveitis (28%). Among uveitis cases, one complete VKH syndrome and one birdshot retinochoroidopathy were identified; other irAEs included ocular surface disorders, optic neuropathy, and inflammatory orbitopathy [3].

Our case documents a combination of posterior segment involvement (chorioretinal lesions) and orbital inflammation that is highly suggestive of a multifocal immune-mediated ocular process triggered by pembrolizumab. Pembrolizumab is a humanized monoclonal antibody directed against PD-1. It is indicated for the treatment of multiple malignancies, including unresectable or metastatic cutaneous melanoma [2].

Several case reports have documented pembrolizumab-associated ocular irAEs, highlighting diverse phenotypes and reinforcing the need for ophthalmologic vigilance during therapy. These have included corneal ulceration with ocular surface inflammation [6], birdshot-like chorioretinopathy [7], panuveitis and retinal vasculitis [8] and optic neuropathy [9]. Although uncommon, these cases illustrate that pembrolizumab can trigger inflammatory responses across virtually all ocular tissues.

Notably, posterior segment involvement, including choriocapillaritis, white dot syndrome-like presentations, and immune-mediated retinopathies, has been increasingly recognized in association with PD-1 inhibition [3,5-10]. In this context, Acaba-Berrocal et al. reported a patient with metastatic cutaneous melanoma who developed an HLA-A29-negative birdshot-like chorioretinopathy approximately two years after the initiation of pembrolizumab, further expanding the spectrum of ICI-related choroidal inflammation [7]. Our case adds to these observations by illustrating multifocal choriocapillaritis associated with orbital inflammatory disease under pembrolizumab, thereby reinforcing the need to consider immune checkpoint inhibitor toxicity in atypical posterior uveitis and chorioretinal presentations.

Our patient developed bilateral pigmentary chorioretinal changes in the posterior pole and periphery with hypo-autofluorescent spots on FAF, while ICGA revealed persistent hypercyanescence foci alongside punctate hypocyanescent areas, consistent with choriocapillaris hypoperfusion and inflammatory choroidopathy. This pattern has been described in ICI-associated white dot-like syndromes and autoimmune retinopathies [3-7]. FAF and OCT remained largely preserved centrally, with no macular oedema or photoreceptor disruption, explaining the patient’s maintained visual acuity (10/10 OS, 6/10 OD due to pterygium-related astigmatism rather than retinopathy). The absence of outer retinal loss or macular involvement is an important prognostic factor, as structural macular changes often predict poorer visual outcomes in ICI-related retinopathies [3-5].

MRI demonstrated fusiform enlargement of multiple extraocular muscles with dilated and thickened muscle bellies, T2 hyperintensity, heterogeneous post-contrast enhancement, and inflammatory infiltration of intraconal fat, while sparing the tendinous insertions and showing no exophthalmos or optic nerve compression. This constellation strongly suggests ICI-associated orbital inflammation, a rare but increasingly reported orbital irAEs [3-4]. The absence of optic neuropathy is a crucial element supporting the decision for conservative management.

The coexistence of choriocapillaritis and orbital inflammation in this patient illustrates the broad range of ocular tissues susceptible to T-cell-mediated injury during ICI therapy. Because melanoma can metastasize to the choroid or orbit, distinguishing autoimmune inflammation from infiltrative disease is essential. Multimodal imaging (optical coherence tomography (OCT), fundus autofluorescence (FAF), indocyanine green angiography (ICGA), and magnetic resonance imaging (MRI) played a central role in excluding metastasis and confirming an inflammatory irAE.

Management of ocular irAEs is challenging because available evidence is limited and therapeutic decisions must balance ocular safety with oncologic benefit [10-12]. In this context, several factors guided the conservative strategy: the patient had extensive metastatic disease at baseline, an excellent systemic response on recent PET-CT with no active disease, and pembrolizumab remained the only effective oncologic option. Ocular involvement was moderate, vision-sparing, and without macular or optic nerve compromise. Therefore, after multidisciplinary discussion, the team elected to continue pembrolizumab while initiating local corticosteroids and implementing close ophthalmologic surveillance, reserving systemic corticosteroids or treatment interruption for worsening inflammation.

This approach is consistent with current irAEs management frameworks, which allow continuation of ICIs in most grade 1-2 events when vision is preserved, and inflammation is manageable with local treatment [11,12]. Treatment cessation is generally recommended only for severe, vision-threatening, or steroid-refractory cases. Current oncology guidelines recommend continuing immune checkpoint inhibition with close monitoring for most grade-1 toxicities, reserving suspension for higher-grade or life-threatening events [11].

In the ophthalmic literature, several papers have highlighted that checkpoint inhibitor-associated uveitis and other ocular irAEs respond well to topical or systemic corticosteroids, and do not necessarily mandate discontinuation of immunotherapy, particularly in non-vision-threatening cases [10-12].

Previous studies have reported that local treatment alone can effectively control mild ocular irAEs, and discontinuation of the checkpoint inhibitor is not routinely required. When feasible, local therapy is preferred so that the ICI can be maintained, given the potential adverse effects of prolonged systemic corticosteroid exposure. In addition, the impact of systemic corticosteroids on the efficacy of immunotherapy remains uncertain; therefore, systemic steroids should be avoided when inflammation can be adequately controlled with local treatment [4,11,12].

Most published cases of ICI-related choroidopathy and orbital myositis improve with timely intervention [3,4]. In our patient, the absence of macular injury and the stability of the optic nerve suggest a favourable prognosis for long-term visual preservation. Although no standardized imaging schedule exists, periodic orbital MRI, typically every 6-12 months, or sooner if symptoms recur, is reasonable for monitoring.

## Conclusions

Immune checkpoint inhibitors such as pembrolizumab may induce ocular immune-related adverse events that require careful clinical judgment. This case highlights the complexity of managing ICI-associated inflammation, particularly when it presents with unusual patterns like simultaneous choriocapillaris involvement and orbital inflammation. Despite its atypical presentation, the inflammation remained mild with preserved vision and excellent systemic control. These features allowed continuation of pembrolizumab with local therapy, demonstrating that even unusual ocular irAEs do not necessarily require stopping immunotherapy when severity is limited. As the use of ICIs expands, greater awareness and timely management of ocular irAEs are critical to prevent irreversible visual morbidity while maintaining optimal oncologic outcomes.
